# Supplementary material for: Simulated redistricting plans for the analysis and evaluation of redistricting in the United States
Source: Sci Data. 2022 Nov 11;9:689. doi: 10.1038/s41597-022-01808-2 (PMC9652457; doi:10.1038/s41597-022-01808-2)
Supplement: Supplementary file 1 — Supplementary Information [file 41597_2022_1808_MOESM1_ESM.pdf]

Supplementary Information: Simulated redistricting plans for the analysis and evaluation of redistricting in the United States

October 24, 2022

Contents

1 Table of State Rules **2**

1 Table of State Rules

Redistricting rules vary across states. However, all states require districts to be contiguous and have approximately equal populations. Further, many states include references to avoid splitting particular geographic areas, such as counties or "communities of interest." As a result, we use a set of common "base constraints" applied to every state, and incorporate additional constraints for particular state circumstances (e.g., Colorado requires competitive districts, Louisiana requires preserving the cores from previous districts, etc.).

Our base constraints are contiguity, approximately equal populations, and a county / municipality constraint to avoid splitting. Further, districts are drawn to be compact and are compared with the metrics described in the main text, unless the state explicitly mentions a measure that should be used (e.g., Iowa). Consistent with laws in many states, none of our districts are drawn to favor incumbents or a political party.

Some states, such as Virginia and Pennsylvania, have residential segregation by race which naturally ensures that minority opportunity districts are included from a race-blind simulation process. While we check performance as a validation step, no additional constraints are needed within the simulations to ensure that compliance.

State	Legal Criteria (beyond Federal)	Constraints
**Alabama**	Reapportionment Committee<br>Redistricting Guidelines	- Base constraints<br>- VRA constraint (BVAP)
**Arizona**	State Constitution (Article 4)	- Base constraints<br>- Favor competitive districts<br>- VRA constraint (HVAP)
**Arkansas**	No additional laws	- Base constraints
**California**	California State Constitution (Article 21)	- Base constraints<br>- VRA constraints (HVAP, BVAP, AVAP)
**Colorado**	Colorado State Constitution (Section 44)	- Base constraints<br>- Favor competitive districts
**Connecticut**	No additional laws	- Base constraints
**Florida**	Florida State Constitution<br>(Article 3, Section 20)	- Base constraints<br>- VRA constraint (BVAP and HVAP)
**Georgia**	2021-22 Guidelines for the<br>House Legislative and<br>Congressional Reapportionment Committee	- Base constraints<br>- VRA constraint (BVAP)
**Hawaii**	HRS Title 1, S25	- Base constraints<br>- Census tracts not split<br>- Complete district in Honolulu
**Idaho**	Idaho State Constitution<br>Title 72, Chapter 15	- Base constraints<br>- Connect counties based on highways
**Illinois**	Illinois State Constitution<br>Article 4, Section 3	- Base constraints<br>- VRA constraint (BVAP)
**Indiana**	Indiana State Constitution	- Base constraints
**Iowa**	Iowa State Constitution<br>Article 3	- Base constraints<br>- Districts constructed from counties<br>- Be compact, as defined by length-width and perimeter compactness

State	Legal Criteria (beyond Federal)	Constraints
**Kansas**	Proposed Guidelines and Criteria for 2022 Kansas Congressional Redistricting	- Base constraints<br>- Preserve the cores of existing districts
**Kentucky**	Criteria and Standards for Congressional Redistricting adopted by Interim Joint Committee on State Government's Redistricting Subcommittee (1991)	- Base constraints
**Louisiana**	Louisiana Joint Rule No. 21	- Parish and municipality constraint<br>- Preserve cores of previous districts<br>- VRA constraint (nonwhite VAP)
**Maine**	Title 21A, Chapter 16, Section 1206	- Base constraints
**Maryland**	Maryland State Constitution (Article 3)<br>Executive Order 01.01.2021.02	- Base constraints
**Massachusetts**	Massachusetts State Constitution (Articles XVI, CI)	- Base constraints
**Michigan**	Michigan State Constitution (Article 4)	- Base constraints<br>-VRA constraint (nonwhite VAP)
**Minnesota**	Minnesota State Constitution (Article 4)<br>Minnesota 2021 Statute 2.91	- Base constraints
**Mississippi**	Mississippi State Constitution (Article 3)<br>Agreements of the State Legislature Joint Legislative Committees on Reapportionment and Redistricting	- Base constraints<br>- VRA constraint (BVAP)
**Missouri**	Missouri State Constitution (Article 3)	- Base constraints<br>- VRA constraint (BVAP)
**Montana**	Montana State Constitution (Article 5)	- Base constraints
**Nebraska**	Nebraska State Constitution (Article 3)<br>Legislative Resolution 134	- Base constraints<br>- Preserve cores of previous districts
**Nevada**	Nevada State Constitution (Article 4)	- Base constraints
**New Hampshire**	New Hampshire State Constitution (Part 2)	- Base constraints<br>- Townships not split
**New Jersey**	New Jersey State Constitution (Articles 2, 4)	- Base constraints
**New Mexico**	New Mexico State Constitution (Article 4), SB304	- Base constraints<br>- Preserve cores of previous districts
**New York**	New York State Constitution (Article 3)	- Base constraints
**North Carolina**	North Carolina State Constitution (Article 2)	- Base constraints<br>- VRA constraint (BVAP)
**Ohio**	Ohio State Constitution (Article XIX)	- Base constraints<br>- Not split Cincinnati or Cleveland<br>- Minimize splitting of Columbus<br>- Split no more than 18 counties once, and no more than 5 counties twice, and no counties three times

State	Legal Criteria (beyond Federal)	Constraints
**Oklahoma**	Oklahoma State Constitution (Article 5)<br>2021 Senate Select Committee<br>On Redistricting Guidelines	- Base constraints
**Oregon**	Oregon State Constitution (Article 4)<br>Revised Statute 188.010	- Base constraints<br>- Districts must be connected by transportation links
**Pennsylvania**	Pennsylvania State Constitution (Article 2)	- Base constraints
**Rhode Island**	Rhode Island State Constitution<br>Public Laws, Chapter 100, Section 2	- Preserve state senate districts<br>- VRA constraint (nonwhite VAP)
**South Carolina**	Guidelines and Criteria for Congressional and Legislative Redistricting	- Base constraints<br>- VRA constraint (BVAP)
**Tennessee**	No additional laws	- Base constraints
**Texas**	No additional laws	- Base constraints<br>- VRA constraint (HVAP and BVAP)
**Utah**	Utah State Constitution (Article 9)<br>Code 20A-20-302	- Base constraints<br>- Preserve cores of prior districts
**Virginia**	Virginia State Constitution (Article 2)<br>Code 24.2-304.04	- Base constraints
**Washington**	Washington State Constitution (Article 2)<br>RCW 44.05.090	- Base constraints<br>- Not be connected across geographic barriers, although ferries across water may establish contiguity - VRA constraint (nonwhite VAP)
**West Virginia**	West Virginia State Constitution (Article 1)	- Base constraints<br>- Counties not split
**Wisconsin**	Wisconsin State Constitution (Article 4)	- Base constraints

Table 1: Redistricting rules incorporated in our simulations for all states with redistricting processes. All states include a set of "base" constraints that include contiguity, approximately equal populations, and a county / municipality constraint to avoid splitting. Further, we implement state-specific criteria when available. More detail on specific code implementation of these constraints is available on our website, https://github.com/alarm-redist/fifty-states.
